# Modulation of stress granules by lobeline increases cell death in hypoxia and impacts the ability of glioblastoma cells to secrete extracellular vesicles

**DOI:** 10.1038/s41420-025-02692-6

**Published:** 2025-10-06

**Authors:** Kathleen M. Attwood, Lauren P. Westhaver, Aaron Robichaud, Jae Ho Han, Sydney Croul, Gabriel Wajnberg, Jeremy W. Roy, Adrienne C. Weeks

**Affiliations:** 1 Dalhousie University Department of Surgery, Halifax, NS Canada; 2 Dalhousie University Division of Neurosurgery, Saint John, NB Canada; 3 Dalhousie University Division of Neurosurgery, Halifax, NS Canada; 4 Dalhousie University Department of Medical Neuroscience, Halifax, NS Canada; 5 Dalhousie University Department of Pathology, Halifax, NS Canada; 6 Atlantic Cancer Research Institute, Moncton, NB Canada

**Keywords:** CNS cancer, Mechanisms of disease

## Abstract

Glioblastoma (GBM) is a devastating universally fatal primary brain cancer. Novel therapeutic strategies are required to alter disease course and improve survival in these patients. There is increasing evidence that modulating cancer’s ability to respond to and survive cellular stress through RNA stress granules (SGs) may be a novel approach to cancer therapeutics. SGs are cytoplasmic aggregates of untranslated mRNAs and RNA binding proteins formed in response to a variety of cellular stressors, that allow cells to temporarily prioritize translation of stress-related proteins. A previous drug screen identified the dopamine modulator lobeline as a factor affecting SG disassembly in GBM cells. Lobeline impairs GBM cell survival by impairing SG disassembly after hypoxia. Specifically, after a hypoxic challenge, lobeline “locks” cells in a stressed state, even after re-exposure to normoxia. This is characterized by retained SGs, elevated levels of phosphorylated eIF2α and a sustained reduction in global protein translation. The disruption of the canonical stress response induced by lobeline ultimately results in increased cell death in both primary and immortalized GBM cell lines. Interestingly, lobeline also reduces post-hypoxia extracellular vesicle (EV) release, potentially through sequestration of the SG and EV protein, YBX1. Taken together, this adds to the literature that modulating stress and SG dynamics may be useful alone or to potentiate other treatment modalities affecting stress in GBM.

## Introduction

Glioblastoma (GBM) is the most common and aggressive (Grade IV; IDH wildtype) primary malignant brain tumor. Despite standard treatment, which includes maximal surgical resection followed by adjunctive chemotherapy (temozolomide) and radiation, GBM is universally fatal with a median survival of ~15 months following diagnosis [1, 2]. This can be attributed to GBM’s aggressive growth pattern, adaptability, diffuse infiltration, and genetic heterogeneity, all of which lead to high recurrence rates [3]. Due to the poor prognosis of GBM, there is a pressing need to investigate novel therapeutic approaches that address these challenges.

Rapidly growing tumor cells are exposed to a variety of adverse microenvironmental conditions, such as nutrient deprivation and hypoxia. Hypoxia is a hallmark of GBM and is correlated with enhanced tumor aggressiveness and therapeutic resistance [4–6]. In response to various stressors, including hypoxia, cells trigger a conserved adaptive process known as the integrated stress response (ISR) to ensure survival until the stress condition is ameliorated and homeostasis is restored. Four stress sensing kinases respond to various forms of cellular stress and converge on eukaryotic translation initiation factor 2 subunit alpha (eIF2α), phosphorylating the key translation initiation factor, ultimately resulting in translation inhibition [7]. During the ISR, with global protein translation suppressed, untranslated mRNA transcripts and aggregation-prone RNA binding proteins such as Ras GTPase-activating protein-binding protein 1/2 (G3BP1/2) and T-cell-restricted intracellular antigen-1 related protein (TIAR) are packaged into non-membrane bound foci known as stress granules (SGs) [8]. Co-localization of transcripts and translational machinery in SGs allows cells to prioritize translation of stress-related genes and sequester apoptotic proteins facilitating cellular survival [8–10].

SGs are dynamic structures and can be disassembled within minutes to hours upon stress removal [11, 12]. The phosphorylation of eIF2α can be reversed by the action of one of two key phosphatases: the constitutively expressed constitutively active reverter of eIF2α phosphorylation (CReP) or by the stress-induced phosphatase, growth arrest and DNA damage-inducible protein (GADD34) [13], allowing for protein translation to resume. SG dissolution is then thought to occur through a multistep process involving post-translational modifications such as ubiquitination and SUMOylation that ultimately results in clearance through autophagy [14–16]. Excessive stressors that overwhelm cellular adaptation can trigger the activation of pathways leading to regulated cell death (apoptosis) [17]. Immunohistochemical labeling of a human GBM tissue microarray (*n* = 90) previously published by our laboratory demonstrated the formation of SGs adjacent to areas of necrosis, reflecting their relevance in the in vivo GBM tumor microenvironment [18].

A high-throughput screen for compounds that would alter hypoxia-induced SG dynamics in GBM cells previously identified the selective estrogen receptor modulator (SERM) raloxifene as preventing SG dissolution following hypoxic stress release, resulting in cell death [18]. Lobeline, a drug that can interact with nicotinic receptors, is a vesicular monoamine transporter 2 (VMAT2) ligand, and can stimulate dopamine storage and release [19, 20] was also identified in this screen (Table 1). Lobeline has not been investigated in GBM but has been shown to reverse drug resistance in cancer cells and to decrease colon cancer growth [21, 22].

**Table 1:** Top 10 drug candidates that prevent SG dissolution in GBM cells post hypoxia.

Drug	Drug class	*Z* score	Retained SGs after hypoxia?	Increased SG formation?
Chelidonine (+)	Monoamine Metabolism	47.59765484	Yes	Yes*
Scoulerine	Monoamine Metabolism	40.01073732	Yes	Yes*
Piperlongumine	Oxidative Stress	19.40302893	Yes	Yes*
**Lobeline alpha (−) hydrochloride**	**Monoamine Metabolism**	**17.04493294**	**Yes**	**No**
Benzethonium chloride	Antimicrobial	10.62192853	Yes	No
Metergoline	Monoamine Metabolism	10.4904695	Yes	No
Nortriptyline hydrochloride	Monoamine Metabolism	10.42626503	Yes	No
Lobelanidine hydrochloride	Monoamine Metabolism	10.38074863	Yes	No
Benzamil hydrochloride	Electrolyte Homeostasis	9.980604543	Yes	No
Desipramine hydrochloride	Monoamine Metabolism	8.927833286	Yes	No
Raloxifene hydrochloride	Estrogen Receptor Modification	4.894554443	Yes	No

*Originally excluded from consideration as potential false positive; may drive SG formation rather than prevent SG dissolution.

1120 FDA approved drugs (Prestwick Chemical Library) were previously screened for their impact on SG dynamics in GBM cells. The top 10 drug candidates that delayed SG dissolution relative to vehicle controls 1 h post-hypoxia are listed along with their drug class. Raloxifene hydrochloride (within top 30 drug candidates according to *Z* score) was initially investigated due to SERMs prior uses as auxiliary therapeutics in GBM.

Intriguingly, our laboratory has identified microRNA categories related to both dopamine metabolism and nicotinic acetylcholine receptor signaling pathways significantly upregulated in extracellular vesicle (EV) cargo isolated from GBM patient plasma (Han et al., manuscript in review). Communication between malignant cells is critical for the initiation and progression of cancer. Such intercellular communication can occur via secretion of EVs, which can modulate the tumor microenvironment to promote cancer cell growth and survival [23, 24]. Recent literature has suggested that crosstalk occurs between EVs and SGs. Hypoxia increases EV secretion [25–28], modifies EV cargo [29], and secreted EVs affect the ability of recipient cells to respond to stress utilizing SGs [30]. This may be in part due to Y box binding protein 1 (YBX1), which has a dual role in SGs and EVs [31–33].

Lobeline was identified as a modulator of both SG disassembly and EV secretion during hypoxia induced stress in GBM cells. By inhibiting SG dissolution, lobeline offers a novel therapeutic approach to counteract the stress-adaptive mechanisms of GBM. Additionally, the relationship between SGs and EV secretion highlights a complex network of intercellular communication that may facilitate tumor progression.

## Results

### Lobeline alters SG dynamics by delaying SG dissolution post-hypoxia in immortalized and primary GBM cells

**Fig. 1:**
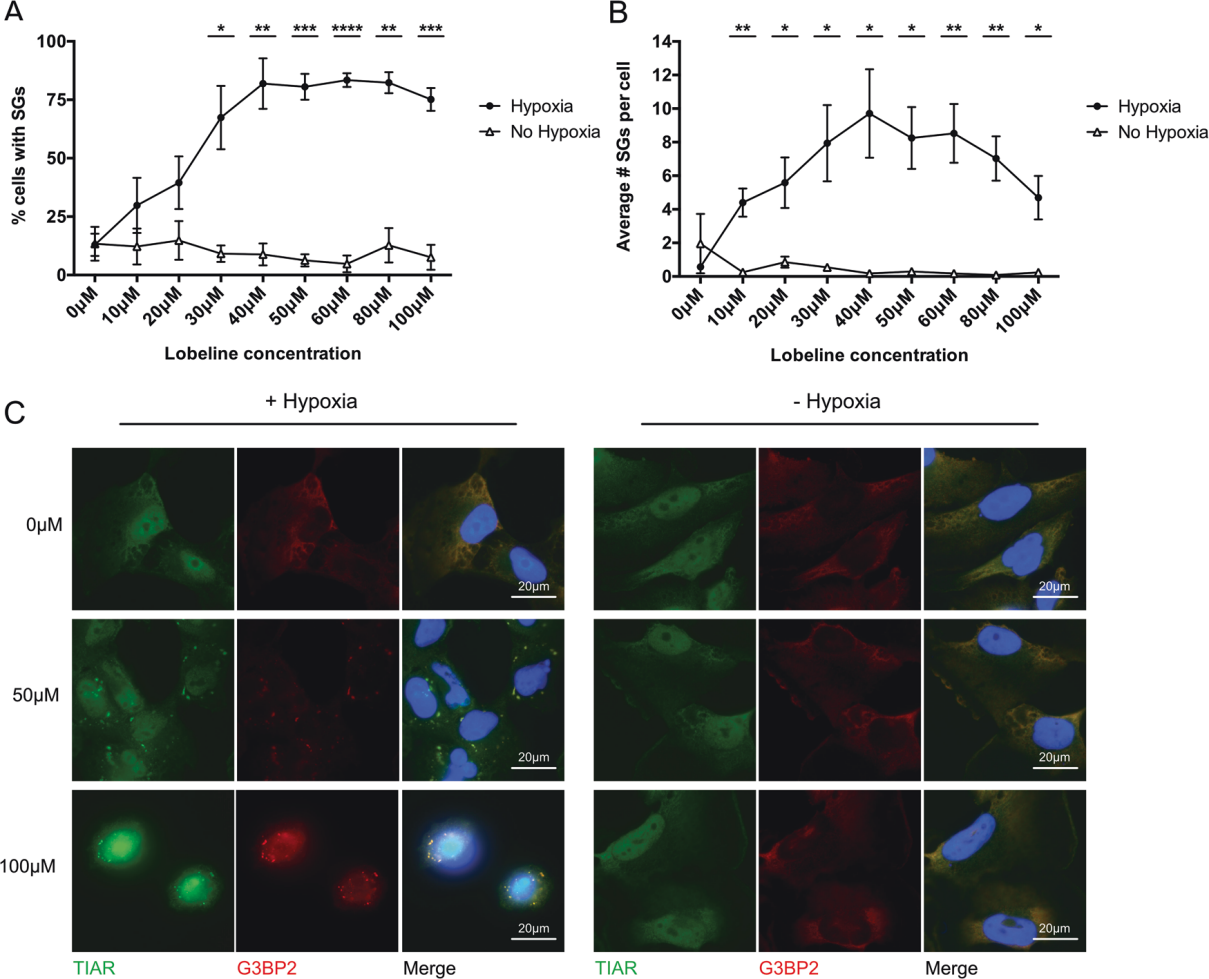
Increasing lobeline concentrations leads to a corresponding increase in retained SGs post-hypoxia. **A**, **B** Human immortalized GBM U251 cells were treated with increasing concentrations of lobeline (0–100 μM) for 1 h prior to a 2 h incubation ± hypoxia (<1% O_2_). Cells were allowed to recover for 30 min before being fixed and stained for cellular membranes (WGA), SGs (TIAR and G3BP2) and nuclei (DAPI). Images were processed through a CellProfiler automated pipeline for quantification of **A** percent cells with SGs and **B** average number of SGs per cell (in those cells with SGs) based on correlative TIAR and G3BP2 immunostaining. Data are presented as the mean of biological replicates (*N* = 3) ± SEM, multiple unpaired *t*-tests *p < 0.05, **p < 0.01, ***p < 0.001, ****p < 0.0001. **C** Representative immunofluorescence of U251 cells (from **A**, **B**) treated with 0, 50 and 100 μM lobeline ± hypoxia.

A previous drug screen identified several FDA approved compounds that altered SG dynamics in immortalized U251 GBM cells exposed to hypoxia [18], with lobeline being the fourth highest candidate by *Z*-score to delay SG dissolution (Table 1). This dissolution was found to be dose dependent, as higher lobeline concentrations resulted in corresponding increases in the percentage of cells containing SGs and the average number of SGs remaining per cell post-hypoxia (Fig. 1A, B). Lobeline reached its effective peak on SG dissolution at 40–60 μM, as higher concentrations caused sustained morphological changes (cell rounding and shrinking) and SG staining became less distinctive with decreased cytoplasmic volume (Fig. 1C, lower panel, 100 μM). Importantly, lobeline did not induce SGs in normoxia at any tested concentration (Fig. 1C, and data not shown).

**Fig. 2:**
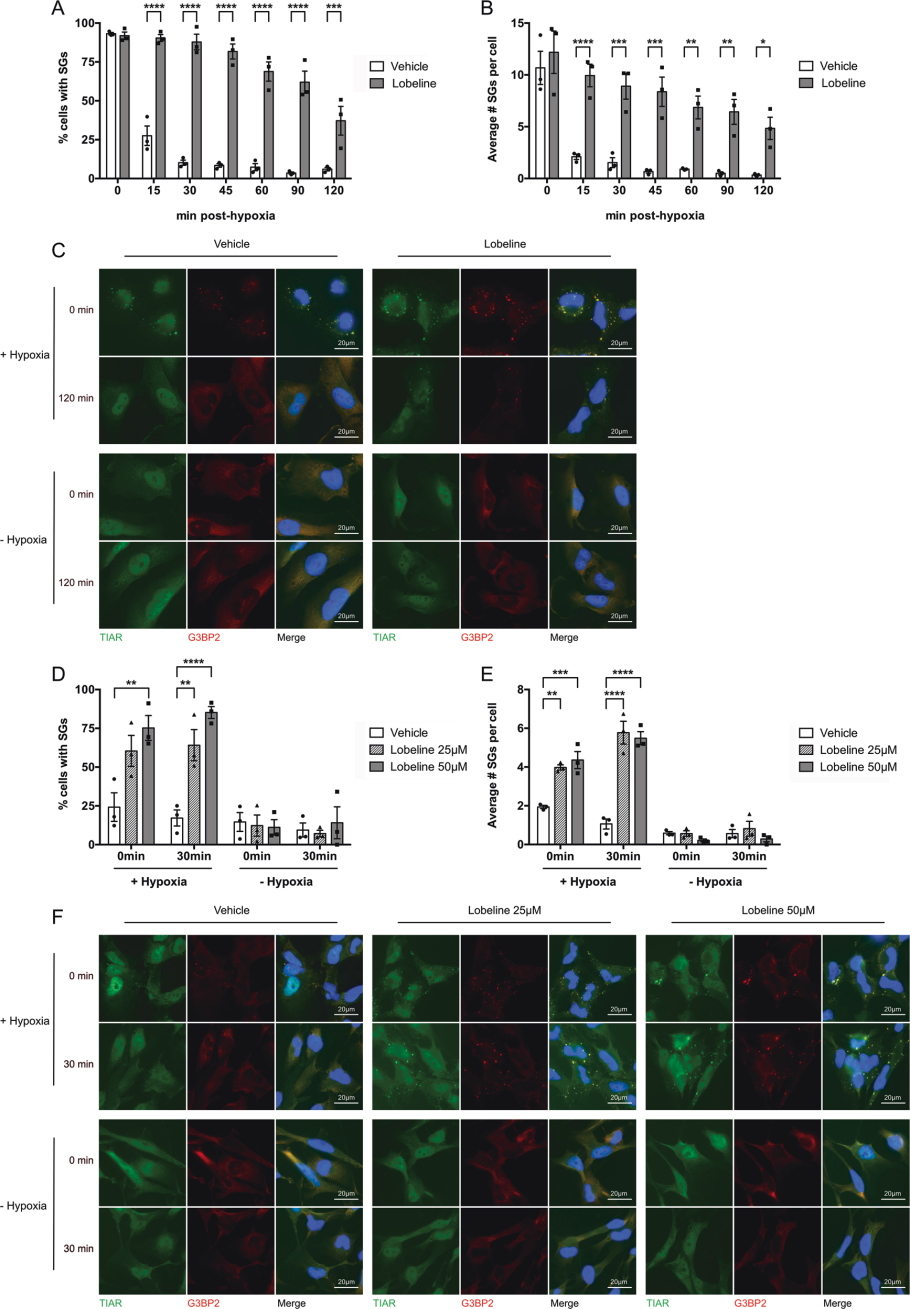
Lobeline delays SG dissolution post-hypoxic release in both immortalized and primary human GBM cells. **A**, **B** Human immortalized U251 GBM cells were treated with 50 μM lobeline or vehicle control for 1 h prior to a 2 h hypoxic incubation (<1% O_2_). At various times post-hypoxia (0–120 min) cells were fixed and stained for cellular membranes (WGA), SGs (TIAR and G3BP2) and nuclei (DAPI). Images were processed through a CellProfiler automated pipeline for quantification of **A** percent cells with SGs and **B** average number of SGs per cell (in those cells with SGs) based on correlative TIAR and G3BP2 immunostaining. Data are presented as the mean of biological replicates (*N* = 3) ± SEM, two-way ANOVA, Sidak’s multiple comparisons test *p < 0.05, **p < 0.01, ***p < 0.001, ****p < 0.0001. **C** Representative immunofluorescence of U251 cells from **A**, **B** at T = 0 min or 120 min ± hypoxia. **D**, **E** Human primary U3085 GBM cells were treated with either 25 μM or 50 μM lobeline or vehicle control (matched to 50 μM concentration) for 1 h prior to a 1 h incubation ± hypoxia (<1% O_2_). Cells were then fixed immediately (0 min) or after 30 min before being fixed and stained (as in **A**, **B**) for automated CellProfiler quantification of **D** percent cells with SGs and **E** average number of SGs per cell (in those cells with SGs). Data are presented as the mean of biological replicates (*N* = 3) ± SEM, two-way ANOVA, Tukey’s multiple comparisons test **p < 0.01, ***p < 0.001, ****p < 0.0001. **F** Representative immunofluorescence of U3085 cells from **D**, **E** at T = 0 min or 30 min ± hypoxia.

In U251 cells, hypoxia-induced SGs typically dissolve within 15–30 min upon return to normoxia (Fig. 2). However, pre-treatment with lobeline was found to significantly delay SG dissolution up to 2 h post-hypoxic release. The percentage of lobeline-treated cells containing SGs as well as the average number of SGs per cell remained significantly higher than untreated controls, with 19.5–50.7% of cells still containing an average of 3–6.7 SGs after 2 h in normoxia (Fig. 2A, B). Notably, lobeline did not induce SG formation in normoxia (Fig. 2C). This delayed SG dissolution was also confirmed in a primary GBM cell line U3085. U3085 cells appear to exhibit some resistance to hypoxia as only 12.3–42.3% of control cells formed SGs immediately post-hypoxic release, and these cells only contained an average of 2 SGs per cell (Fig. 2D, E). Thirty minutes post-hypoxic release, U3085 control cells were able to dissolve the few SGs they contained (Fig. 2D, E). Interestingly, when U3085 cells were incubated with increasing lobeline concentrations, the percentage of cells containing SGs, as well as the average number of SGs per cell significantly increased immediately post-hypoxia. U3085 cells were unable to dissolve these SGs after 30 min in normoxia with 50.8–83.8% and 78.1–91.5% of cells still containing SGs after having been pre-treated with 25 μM and 50 μM of lobeline respectively (Fig. 2D). Lobeline alone at either concentration did not induce SG formation in U3085 cells in normoxia (Fig. 2F).

### Lobeline-induced delays in SG dissolution correspond with decreased protein translation and sustained ISR activation

**Fig. 3:**
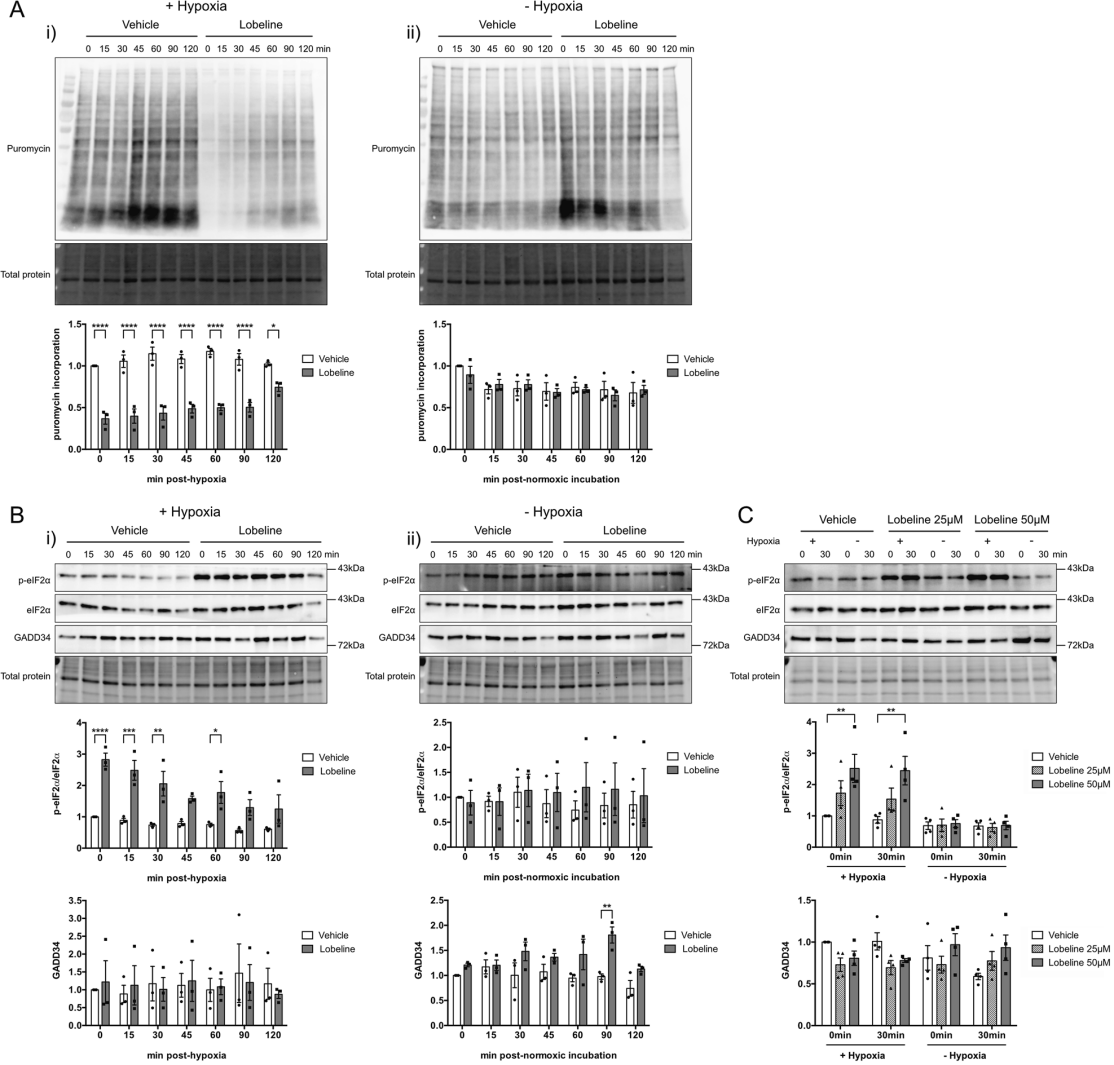
Lobeline prolongs the restoration of protein synthesis and the phosphorylation of eIF2α post-hypoxia. **A** U251 cells were treated with 50 μM lobeline or vehicle control for 1 h prior to a 2 h incubation ± hypoxia (<1% O_2_). At various times post-hypoxic or corresponding normoxic incubation (0–120 min) cells were treated with puromycin and total cell lysates harvested. Puromycin incorporation into nascent polypeptide chains of cells that received hypoxia (i) or remained in normoxia (ii) was detected by anti-puromycin western blot. **B** U251 cells were treated as in **A** but without puromycin treatment. Total cell lysates were harvested and probed for total eIF2α, phospho-eIF2α and GADD34 at various times (0–120 min) post hypoxic (i) or normoxic (ii) incubation. **C** U3085 cells were treated with either 25 μM or 50 μM lobeline or vehicle control (matched to 50 μM concentration) for 1 h prior to a 1 h incubation ± hypoxia (<1% O_2_). Total cell lysates were harvested immediately (0 min) or after 30 min and probed for total eIF2α, phospho-eIF2α and GADD34. All blots were normalized to their respective total lane protein and are represented as ratios with vehicle control T = 0 min normalized to 1. The level of eIF2α phosphorylation is presented as the ratio of phospho-eIF2α to total eIF2α. Data are presented as the mean of biological replicates (*N* = 3 U251; *N* = 4 U3085) ± SEM, two-way ANOVA, Sidak’s (**A**, **B**) or Tukey’s (**C**) multiple comparisons test *p < 0.05, **p < 0.01, ***p < 0.001, ****p < 0.0001.

Lobeline treatment in U251 cells was associated with sustained eIF2α phosphorylation after a return to normoxia compared to vehicle treated controls (Fig. 3Bi–ii). As expected, with sustained eIF2α phosphorylation cells demonstrated a decrease in global protein translation as determined by a puromycin incorporation assay (Fig. 3Ai). The effect of lobeline lasted in excess of 2 h after removal from hypoxia despite global translation returning to normal within 15 min in vehicle control treated cells (Fig. 3Ai). This was not due to lobeline directly influencing protein translation, as lobeline alone had no impact on the level of puromycin incorporation in normoxia (Fig. 3Aii). Lobeline alone also had no effect on eIF2α phosphorylation in normoxia (Fig. 3Bii) and no significant changes in GADD34 protein levels in lobeline-treated cells ± hypoxia were noted (Fig. 3B).

The sustained eIF2α phosphorylation was confirmed in U3085 cells in which lobeline resulted in increased phosphorylated eIF2α up to 30 min post-hypoxia (Fig. 3C). There was also no observable trend in GADD34 protein levels that could explain the elevated eIF2α phosphorylation (Fig. 3C).

### Lobeline does not impact SG dynamics through alterations in mTOR signaling or inhibition of autophagy

Previous work has demonstrated a down regulation of mammalian target of rapamycin (mTOR) signaling during retention of hypoxia-induced SGs [18, 34]. However, no difference in protein levels was observed in the key p70S6 kinase substrate, ribosomal protein S6 (rpS6), between control or lobeline treated U251 cells post-hypoxia (Supplementary Fig. 1A). A block in autophagy could also result in a defect in SG clearance [35, 36] and is the method of SG retention with the known autophagy inhibitor chloroquine [18]. No distinctive trends in LC3BII protein levels between vehicle control and lobeline treated U251 cells ± hypoxia were found (Supplementary Fig. 1A), and importantly LC3BII levels were much lower in all experimental conditions relative to chloroquine (Supplementary Fig. 1B).

### The combination of lobeline and hypoxia shifts cell death towards late apoptosis/necrosis

**Fig. 4:**
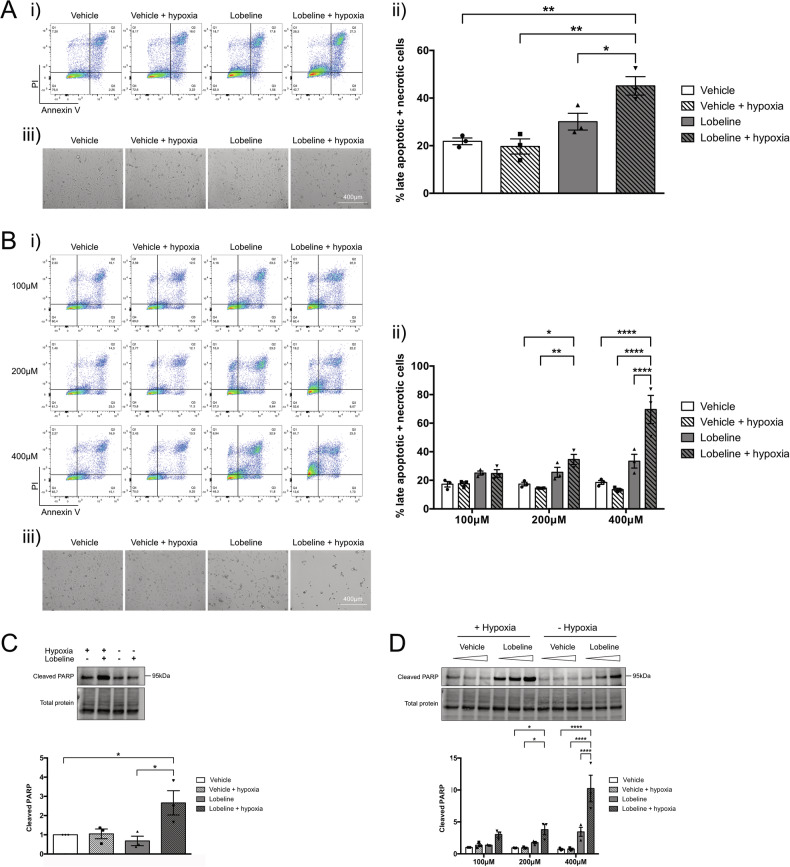
The combination of lobeline and hypoxia leads to synergistic cell death in immortalized GBM cells. **A** U251 cells were treated with 50 μM lobeline for 1 h prior to a 2 h incubation ± hypoxia (<1% O_2_). Cells were allowed to recover for 24 h in normoxia before being harvested and stained live by annexin V/PI and analyzed by flow cytometry. Cells in the lower left quadrant (annexin V−/PI−) were classified as live, the lower right quadrant (annexin V+/PI−) as early apoptotic, the upper right quadrant (annexin V+/PI+) as late apoptotic and the upper left quadrant (annexin V−/PI+) as necrotic (i). The percentage of late apoptotic/necrotic cells were combined and presented graphically as the mean of biological replicates (*N* = 3) ± SEM, one-way ANOVA, Tukey’s multiple comparisons test *p < 0.05, **p < 0.01 (ii). Representative brightfield microscopy of vehicle control and lobeline treated cells ± hypoxia (iii). **B** U251 cells were treated as in **A** except with increasing lobeline concentrations (100–400 μM) and a recovery period of 4 h. Data are presented as the mean of biological replicates ± SEM, two-way ANOVA, Tukey’s multiple comparisons test *p < 0.05, **p < 0.01, ****p < 0.0001. **C**, **D** U251 cells were treated as in **A** or **B** with total cell lysates being harvested and probed for cleaved PARP. Blots were normalized to their respective total lane protein and are represented as ratios with vehicle control (**C**) and vehicle control 100 μM (**D**) normalized to 1. Data are presented as the mean of biological replicates (*N* = 3) ± SEM, one-way ANOVA (**C**) or two-way ANOVA (**D**), Tukey’s multiple comparisons test *p < 0.05, ****p < 0.0001.

Our group has previously demonstrated that inhibition of timely SG dissolution leads to increased GBM cell death [18]. Despite the reduced global translation and retained SGs, no increased cell death was found 2 h post-hypoxia in U251 cells by Annexin/PI (data not shown). However, at 24 h post-hypoxia, cells exposed to lobeline and hypoxia demonstrated a significant increase in late apoptosis/necrosis (Q1 + Q2, Fig. 4Ai) compared to cells that had received either lobeline or hypoxia alone (Fig. 4Ai–iii). When U251 cells received higher concentrations of lobeline (up to 400 μM) prior to hypoxic incubation, cell death occurred faster, with significant numbers of late apoptotic/necrotic cells being observed 4 h post-hypoxic release (Fig. 4Bi–iii). Interestingly, while there is a significant combined increase in late apoptosis and necrosis in U251 cells that received both lobeline (low or high concentration) and hypoxia, there appears to be a distinctive shift in the bulk of these cell populations specifically towards necrosis (shifting from Q4 directly to Q1). Increased U251 cell death was also confirmed by western blot for cleaved PARP, a marker of late-stage apoptosis (Fig. 4C, D).

**Fig. 5:**
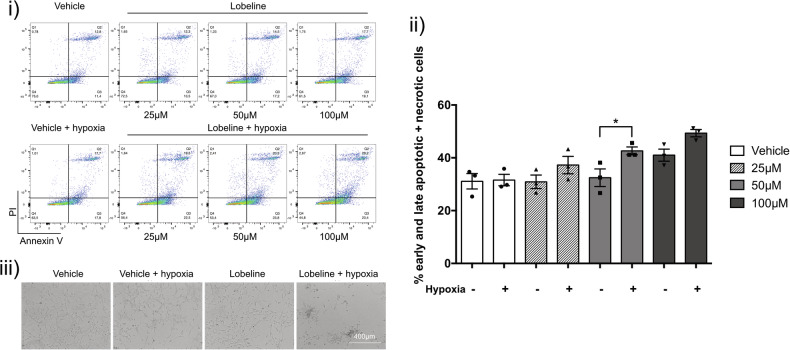
The combination of lobeline and hypoxia leads to synergistic cell death in primary GBM cells. U3085 cells were treated with increasing lobeline concentrations (25–100 μM; vehicle control matched to 100 μM concentration) for 1 h prior to a 2 h incubation ± hypoxia (<1% O_2_). Cells were allowed to recover for 24 h in normoxia before being harvested and stained live by annexin V/PI and analyzed by flow cytometry (i). Data are presented as the mean of biological replicates ± SEM, one-way ANOVA, Sidak’s multiple comparisons test *p < 0.05 (ii). Representative brightfield microscopy of vehicle control and lobeline treated cells ± hypoxia (iii).

Synergistic cell death was also observed in U3085 cells, with a significant increase in early and late apoptosis/necrosis 24 h post-hypoxia in cells that had received 50 μM lobeline with a trend towards significance at higher concentrations (100 μM) (Q1–Q3, Fig. 5).

### Small RNAs from stress-related pathways are over-represented in EVs isolated from human GBM patients as well as miRNA related to dopamine metabolism

Lobeline interacts with both VMAT2 and the dopamine transporter (DAT) and through these interactions can modulate dopamine storage and release [37, 38]. Lobeline also functions as a nicotinic receptor antagonist, inhibiting nicotine-evoked dopamine release [39]. EVs from the plasma of GBM patients had previously been isolated and their small RNA cargo sequenced (Han et al., manuscript in review). Analysis of EV miRNA cargo demonstrates over-representation of categories related to oxidative stress and miRNA functioning in categories related to both dopamine metabolism and nicotinic acetylcholine receptor signaling (Table 2).

**Table 2:** Significantly enriched miRNA categories related to oxidative stress and dopamine metabolism in EVs isolated from high-grade glioma patient plasma.

Category	Subcategory	Enrichment	False discovery rate	Observed mRNA
Stress				
Pathways (miRWalk)	WP408 Oxidative stress	Over-represented	0.0025	10
Pathways (miRWalk)	hsa00620 Pyruvate metabolism	Over-represented	0.004	10
Pathways (miRWalk)	hsa00480 Glutathione metabolism	Over-represented	0.0043	8
Pathways (miRWalk)	P00046 Oxidative stress response	Over-represented	0.0065	11
Pathways (miRWalk)	hsa00270 Cysteine and methionine metabolism	Over-represented	0.0072	9
Pathways (miRWalk)	WP3 Keap1 Nrf2	Over-represented	0.0094	6
Pathways (miRWalk)	WP15 Selenium	Over-represented	0.0126	11
Pathways (miRWalk)	WP28 Selenium metabolism and selenoproteins	Over-represented	0.0146	7
Pathways (miRWalk)	WP314 FAS pathway and stress induction of HSP regulation	Over-represented	0.0149	11
Dopamine metabolism				
Pathways (miRWalk)	hsa00360 Phenylalanine metabolism	Over-represented	0.0013	7
Pathways (miRWalk)	hsa00350 Tyrosine metabolism	Over-represented	0.0025	7
Pathways (miRWalk)	hsa00340 Histidine metabolism	Over-represented	0.0051	6
Pathways (miRWalk)	P02787 Vitamin B6 metabolism	Over-represented	0.0093	4
Pathways (miRWalk)	hsa00750 Vitamin B6 metabolism	Over-represented	0.0146	4
Pathways (miRWalk)	hsa00400 Phenylalanine tyrosine and tryptophan biosynthesis	Over-represented	0.0149	3
Pathways (miRWalk)	P02759 Pyridoxal 5 phosphate biosynthesis	Over-represented	0.0149	3
Pathways (miRWalk)	hsa00380 Tryptophan metabolism	Over-represented	0.0234	6
Pathways (miRWalk)	P00044 Nicotinic acetylcholine receptor signaling pathway	Over-represented	0.0437	8

Significantly enriched oxidative stress and dopamine metabolism-related categories obtained using miEAA 2.0 over-representation analysis for differentially expressed miRNA when comparing EVs of high-grade glioma patients to non-cancer control.

### Lobeline dampens a hypoxic recovery-induced increase in EV secretion

**Fig. 6:**
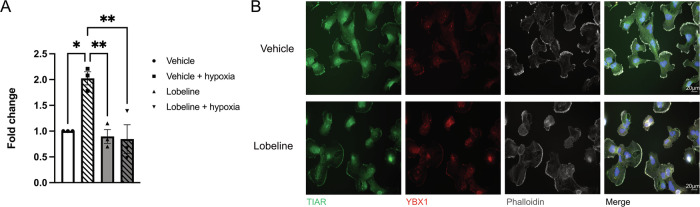
Lobeline dampens hypoxia-induced EV release and sequesters YBX1 in SGs. **A** U251 cells were treated with 50 μM lobeline or vehicle control for 1 h prior to a 2 h incubation ± hypoxia (<1% O_2_). EVs were isolated from conditioned culture media harvested from U251 cells 2 h post hypoxic or corresponding normoxic incubation and quantified by nanoparticle tracking analysis. The average of each biological replicate (*N* = 3) is expressed as a fold change of particles/mL of media ± SEM, one-way ANOVA, Tukey’s multiple comparisons test *p < 0.05, **p < 0.01. **B** Representative immunofluorescence of U251 cells. U251 cells were treated as above, were fixed and stained for actin (phalloidin), SGs (TIAR), YBX1 and nuclei (DAPI) at T = 60 min post-hypoxia.

To determine if there was a link between hypoxia, EV secretory patterns and lobeline, the number of EVs released following a 2-h period post-hypoxia was quantified. Hypoxia is known to increase EV secretion [25–28], and consistent with this, we see a two-fold increase in the amount of EVs released post-hypoxia in vehicle control treated cells relative to cells maintained in normoxia. However, cells pre-treated with lobeline and hypoxic stress have diminished EV secretion compared to cells that received hypoxia alone (Fig. 6A). To understand what could be preventing hypoxia-induced EV release in lobeline treated cells, we immunostained for YBX1. YBX1 is a multifunctional RNA binding protein that plays a role in both SG formation [31] and in selective loading of small non-coding RNAs into EVs [40, 41]. We find that YBX1 is sequestered in SGs of lobeline-treated cells 60 min post-hypoxia. In contrast, YBX1 displays only diffuse cytoplasmic staining in vehicle control treated cells (Fig. 6B).

## Discussion

The poor prognosis of GBM is in part due to its capacity to adapt to and co-opt its surrounding microenvironment, allowing tumors to thrive under adverse conditions. There is growing interest in targeting the stress response, particularly SG formation, for novel cancer therapies [42]. Our previous work demonstrated the presence of cells containing SGs adjacent to the hypoxic GBM core in vivo, in human GBM samples suggesting a role in GBM pathobiology [18]. However, targeting SG formation can be complex, as certain compounds (e.g., glutathione), which inhibit SG formation under hypoxic conditions, also have protective roles in alleviating hypoxic stress, potentially rendering them counterproductive in cancer treatment [18, 43]. This study presents an alternate approach and investigates SG disassembly. This work, together with our previous findings using the drug raloxifene, demonstrates that pharmacologically delaying SG disassembly impairs timely stress resolution and leads to increased GBM cell death. These results further support our hypothesis that targeting SG dissolution represents a valid cancer therapeutic strategy. Here we show that lobeline delays SG dissolution post-hypoxia in a dose-dependent manner, leading to a higher percentage of cells containing SGs, an increased number of SGs per cell, sustained ISR activation and reduced protein synthesis. Furthermore, lobeline in the absence of hypoxia does not result in cell death and thus as a cancer therapeutic may selectively target cancer cells in the hypoxic microenvironment as opposed to normoxic normal cells. Investigating these strategies in an animal model (such as an orthotopic humanized mouse model of GBM) will be an important next step to bring this targeting strategy to clinical fruition.

Impaired SG disassembly and retention is known to play a key role in certain neurodegenerative diseases [44–46]. Although lobeline-treated cells experience granulostasis in conjunction with an active stress response post-hypoxia, it is unlikely that SG persistence itself directly causes the observed increase in GBM cell death. Notably, although some cells retained SGs 2 h post-hypoxia, the majority had initiated SG disassembly, and apoptosis was not detected via Annexin/PI staining (data not shown). Instead, significant levels of late apoptosis/necrosis were observed 4–24 h post-hypoxia during which SGs had already disassembled. While SGs are associated with pro-survival mechanisms including sequestering pro-apoptotic transcripts during the cellular stress response [47, 48], it is conceivable then that SGs would first have to dissolve to release these factors and initiate cell death programs. This would suggest that the inability to timely resolve stress conditions and restore homeostasis, rather than the prolonged presence of SGs pushes cells towards irreversible cell death.

Although the canonical steps in SG assembly are well established (reviewed extensively in [8, 49]), the steps of SG disassembly are less understood. Lobeline does not influence SG dynamics through downregulation of the stress-induced regulator of eIF2α dephosphorylation GADD34, nor does it alter mTOR pathway signaling. Current theories regarding SG disassembly signaling cascades suggest RNP modifications occur, resulting in less RNA-protein and RNA-RNA interactions [50]. This in turn contributes to the dissipation of SGs until they are ultimately cleared by ubiquitination or autophagy [15, 36]. Indeed, raloxifene was found to impair autophagy clearance of SGs in GBM cells [18]. However, we did not observe any distinctive trends in LC3BII levels in lobeline treated cells post-hypoxia, suggesting there are multiple pathways that can interfere with SG disassembly. Recent studies highlight the role of Disassembly Engaged Proteins (DEPs) and SUMOylation in SG disassembly [50, 51] which warrant further exploration to understand how lobeline impairs SG dynamics. Although the method through which lobeline disrupts SG disassembly is unknown, the act of disrupting canonical stress pathway dynamics and preventing timely stress recovery is the important factor. This is evidenced by the fact that we have now identified two different classes of drug, lobeline (monoamine metabolism) and raloxifene (SERM) that clearly function through different mechanisms yet yield the same result: delayed SG disassembly, prolonged ISR activation and increased GBM cell death.

Lobeline a VMAT2 ligand, can modulate dopamine storage and release [37, 38]. Interestingly, in a concurrent study examining miRNA contained within GBM patient plasma EVs, we identified miRNA categories associated with dopamine metabolism that were significantly upregulated compared to normal plasma controls (Han et al., manuscript in review). Notably, 55% of drugs identified in our initial screen, including lobeline, targeted monoamine metabolism [18]. This suggests that neurotransmitter signaling may play an important role in both tumor cell communication and survival under stress conditions. Indeed, existing literature supports cross-talk between EVs, SGs and the ISR [30, 33]. In particular, hypoxia increases EV secretion and alters RNA content of EVs [25–28, 52] and these EVs can then impact stress-naïve cells [30]. We postulated that modulating stress pathway dynamics with lobeline could also alter hypoxia-induced EV release, and indeed lobeline reduced EV secretion under hypoxic conditions compared to vehicle controls. This is exciting as it may represent a novel way in which paracrine signaling could be disrupted in GBM cells. Cellular communication is crucial for tumor survival, and tumor cells use EVs to signal hypoxic conditions to both proximal and distal cell populations [53]. A decrease in EV release, and subsequently EV cargo, could be linked to the retention of YBX1 in SGs in the presence of lobeline. YBX1 is known to mediate sorting of small noncoding RNA into EVs and is a key SG RNP. Therefore, YBX1 sequestration in SGs may limit its availability for EV and EV cargo secretion. However, this does not invalidate other mechanisms such as changes in lysosomal pH during stress [54]. Whether modulation of EV biogenesis post-hypoxia is an important step for SG disassembly, or in this case, a consequence of delayed SG dissolution in the presence of lobeline, requires further investigation.

Here, we conclude that disrupting SG dynamics impairs cellular stress resolution and promotes cell death. Modulating the hypoxic SG response with lobeline represents a promising therapeutic strategy, as lobeline not only increases hypoxia-induced cell death but also potentially disrupts GBM paracrine signaling via EV secretion. Further work in GBM animal models targeting SG dissolution as well as mechanistic cell biology in this field could potentially yield novel therapeutic strategies.

## Materials and methods

### Cell lines and cell culture

U251 MG cells (MilliporeSigma, ECACC, 09063001) were cultured as previously described [18, 55]. U3085 MG (HGCC RRID:CVCL IR95) cells were cultured on polyornithine and laminin coated plates (MilliporeSigma) as per [18, 55]. Cells were cultured at 37 °C with 5% CO_2_ and never allowed to grow beyond 80% confluency. Cells tested negative for mycoplasma.

### Drug treatment and hypoxia

Unless otherwise stated, cells were treated with a concentration of 50 μM (-)-Lobeline hydrochloride (Tocris Bioscience, 134-63-4). U251 cells were seeded at a density of between 75,000–100,000 cells/mL and treated next day with lobeline or vehicle control (dH_2_O) for 1 h at 37 °C. Hypoxia (<1% O_2_, 2 h total) was then induced as previously described [18]. U3085 cells were seeded at a density of between 80,000–100,000 cells/mL and allowed to adhere for 48 h prior to treatment with lobeline/vehicle control in serum-free DMEM (does not contain sodium pyruvate [18]) for 1 h at 37 °C. U3085 cells then received 1 h hypoxia, with the exception of cell death experiments (2 h hypoxia). Cells that did not undergo hypoxia remained in normoxia (20% O_2_) for the same duration as their matched hypoxia counterparts.

### Immunofluorescence

U251 or U3085 cells were fixed and immunostained to allow for automated quantification of the percentage of cells with SGs and the average number of SGs per cell (in those cells with SGs) based on correlative TIAR and G3BP2 immunostaining as previously described [18]. Primary antibodies were used as follows: mouse anti-TIAR (1:200; BD Biosciences, 610352) and rabbit anti-G3BP2 (1:1500; MilliporeSigma, HPA018304). Cells were imaged and SGs quantified by CellProfiler (cellprofiler.org) and RStudio (Supplementary Data) as previously described [18]. For YBX1 immunofluorescence, cells were co-stained with TIAR as above, rabbit anti-YBX1 (1:400; Abcam, ab12148), and Phalloidin 647 (Thermo Fisher Scientific) according to manufacturer’s protocol.

### Puromycin translation assay

Puromycin translation assay was performed as previously described [18].

### Western blot analysis

Total U251 and U3085 cell lysates were harvested as previously described [18]. Primary antibodies were used as follows: mouse anti-puromycin, clone 12D10 (1:6000; MilliporeSigma, MABE343); rabbit anti-eIF2α (1:1000; Cell Signaling Technology, 9722), rabbit anti-phospho-eIF2α (Ser51) (D9G8) XP (1:750; Cell Signaling Technology, 3398), rabbit anti-GADD34 (1:1000; Thermo Fisher Scientific, PA1-139), rabbit anti-cleaved PARP (Asp214) (1:250; Cell Signaling Technology, 9541), rabbit anti-LC3B (1:1000; Cell Signaling Technology, 2775), rabbit anti-S6 ribosomal protein (5G10) (1:10,000; Cell Signaling Technology, 2217), rabbit anti-phospho-S6 ribosomal protein (Ser235/236) (1:10,000; Cell Signaling Technology, 2211). Western blot analysis was performed as previously described [18]. All blots were normalized to their respective total lane protein and band intensities quantified using ImageLab software (Bio-Rad). See Supplementary Data for uncropped western blot images.

### Annexin V/PI cell death assay

U251 cells were treated with either 50 μM lobeline and allowed to recover from hypoxia overnight, or with increasing concentrations (100–400 μM) and allowed to recover for 4 h. U3085 cells were treated with increasing concentrations of lobeline (25–100 μM) and allowed to recover from hypoxia overnight. Vehicle control for U3085 cells was the 100 μM concentration. The Annexin V-Alexa Fluor 488/propidium iodide (PI) dead cell apoptosis kit (Thermo Fisher Scientific V13241) was used to detect early and late apoptosis and necrosis as previously described [18].

### EV isolation and quantification

Media was harvested from U251 cells 2 h post hypoxic or control normoxic incubation and was pre-cleared by centrifugation twice at 3000 × *g* for 15 min. EVs were captured by peptide-affinity (ME-Kit (Urine/Media), BioSynth) following manufacturers’ protocol. EVs were quantified by nano-particle tracking analysis on a Nanosight NS300 (Malvern Panalytical). Briefly, EVs were diluted in water and five videos of 60 s intervals were captured, analyzed with Gain = 512 and shutter at 1300, and averaged.

### Statistical analysis

All statistical analyses were performed using GraphPad Prism (Version 10). Data are presented as the mean of 3 or 4 (as indicated) biological replicates ± SEM with statistical significance being defined as follows: *p < 0.05; **p < 0.01; ***p < 0.001; ****p < 0.0001. Quantitative variables were analyzed using multiple unpaired *t*-tests, one-way ANOVA with either Sidak’s or Tukey’s multiple comparisons test, or two-way ANOVA with either Sidak’s or Tukey’s multiple comparisons test as indicated in figure legends.

## Supplementary information

Supplementary Figure 1

Supplementary Figure 1 Legend

R-Code

Uncropped Westerns

Uncropped Westerns

Uncropped Westerns

Uncropped Westerns

## Data Availability

All data generated or analyzed during this study are included in this published article and its Supplementary Information files.

## References

[1] Stupp R, Mason WP, van den Bent MJ, Weller M, Fisher B, Taphoorn MJ, et al. Radiotherapy plus concomitant and adjuvant temozolomide for glioblastoma. N Engl J Med. 2005;352:987–96. 10.1056/nejmoa043330. 15758009 10.1056/NEJMoa043330

[2] Schaff LR, Mellinghoff IK. Glioblastoma and other primary brain malignancies in adults: a review. JAMA. 2023;329:574–87. 10.1001/jama.2023.0023. 36809318 10.1001/jama.2023.0023 PMC11445779

[3] Campos B, Olsen LR, Urup T, Poulsen HS. A comprehensive profile of recurrent glioblastoma. Oncogene. 2016;35:5819–25. 10.1038/onc.2016.85. 27041580 10.1038/onc.2016.85

[4] Cosse J-P, Michiels C. Tumour hypoxia affects the responsiveness of cancer cells to chemotherapy and promotes cancer progression. Anticancer Agents Med Chem. 2008;8:790–7. 10.2174/187152008785914798. 18855580 10.2174/187152008785914798

[5] Heddleston JM, Li Z, McLendon RE, Hjelmeland AB, Rich JN. The hypoxic microenvironment maintains glioblastoma stem cells and promotes reprogramming towards a cancer stem cell phenotype. Cell Cycle. 2009;8:3274–84. 10.4161/cc.8.20.9701. 19770585 10.4161/cc.8.20.9701 PMC2825672

[6] Chou CW, Wang CC, Wu CP, Lin YJ, Lee YC, Cheng YW, et al. Tumor cycling hypoxia induces chemoresistance in glioblastoma multiforme by upregulating the expression and function of ABCB1. Neuro Oncol. 2012;14:1227–38. 10.1093/neuonc/nos195. 22946104 10.1093/neuonc/nos195 PMC3452342

[7] Pakos‐Zebrucka K, Koryga I, Mnich K, Ljujic M, Samali A, Gorman AM. The integrated stress response. EMBO Rep. 2016;17:1374–95. 10.15252/embr.201642195. 27629041 10.15252/embr.201642195 PMC5048378

[8] Protter DSW, Parker R. Principles and properties of stress granules. Trends Cell Biol. 2016;26:668–79. 10.1016/j.tcb.2016.05.004. 27289443 10.1016/j.tcb.2016.05.004 PMC4993645

[9] Campos-Melo D, Hawley ZCE, Droppelmann CA, Strong MJ. The integral role of RNA in stress granule formation and function. Front Cell Dev Biol. 2021;9:621779. 10.3389/fcell.2021.621779. 34095105 10.3389/fcell.2021.621779 PMC8173143

[10] Das S, Santos L, Failla AV, Ignatova Z. mRNAs sequestered in stress granules recover nearly completely for translation. RNA Biol. 2022;19:877–84. 10.1080/15476286.2022.2094137. 35796440 10.1080/15476286.2022.2094137 PMC9272840

[11] Wheeler JR, Matheny T, Jain S, Abrisch R, Parker R. Distinct stages in stress granule assembly and disassembly. Elife. 2016;5. 10.7554/eLife.18413. 10.7554/eLife.18413 PMC5014549 27602576

[12] Hofmann S, Kedersha N, Anderson P, Ivanov P. Molecular mechanisms of stress granule assembly and disassembly. Biochim Biophys Acta Mol Cell Res. 2021;1868:118876. 10.1016/j.bbamcr.2020.118876. 33007331 10.1016/j.bbamcr.2020.118876 PMC7769147

[13] Dalton LE, Healey E, Irving J, Marciniak SJ. Phosphoproteins in stress-induced disease. Prog Mol Biol Transl Sci.2012;106:189–221. 10.1016/B978-0-12-396456-4.00003-1. 22340719 10.1016/B978-0-12-396456-4.00003-1

[14] Wang B, Maxwell BA, Joo JH, Gwon Y, Messing J, Mishra A, et al. ULK1 and ULK2 regulate stress granule disassembly through phosphorylation and activation of VCP/p97. Mol Cell. 2019;74:742–57. 10.1016/j.molcel.2019.03.027. 30979586 10.1016/j.molcel.2019.03.027 PMC6859904

[15] Gwon Y, Maxwell BA, Kolaitis RM, Zhang P, Kim HJ, Taylor JP. Ubiquitination of G3BP1 mediates stress granule disassembly in a context-specific manner. Science. 2021;372:6548. 10.1126/science.abf6548. 10.1126/science.abf6548 PMC8574224 34739333

[16] Bennett CL, La Spada AR. SUMOylated Senataxin functions in genome stability, RNA degradation, and stress granule disassembly, and is linked with inherited ataxia and motor neuron disease. Mol Genet Genom Med. 2021;9:1745. 10.1002/mgg3.1745. 10.1002/mgg3.1745 PMC8683630 34263556

[17] Galluzzi L, Bravo-San Pedro JM, Vitale I, Aaronson SA, Abrams JM, Adam D, et al. Essential versus accessory aspects of cell death: recommendations of the NCCD 2015. Cell Death Differ. 2015;22:58–73. 10.1038/cdd.2014.137. 25236395 10.1038/cdd.2014.137 PMC4262782

[18] Attwood KM, Robichaud A, Westhaver LP, Castle EL, Brandman DM, Balgi AD, et al. Raloxifene prevents stress granule dissolution, impairs translational control and promotes cell death during hypoxia in glioblastoma cells. Cell Death Dis. 2020;11:989. 10.1038/s41419-020-03159-5. 33203845 10.1038/s41419-020-03159-5 PMC7673037

[19] Hojahmat M, Horton DB, Norrholm SD, Miller DK, Grinevich VP, Deaciuc AG, et al. Lobeline esters as novel ligands for neuronal nicotinic acetylcholine receptors and neurotransmitter transporters. Bioorg Med Chem. 2010;18:640–9. 10.1016/j.bmc.2009.12.002. 20036131 10.1016/j.bmc.2009.12.002 PMC3726004

[20] Crooks PA, Zheng G, Vartak AP, Culver JP, Zheng F, Horton DB, et al. Design, synthesis and interaction at the vesicular monoamine transporter-2 of lobeline analogs: potential pharmacotherapies for the treatment of psychostimulant abuse. Curr Top Med Chem. 2011;11:1103–27. 10.2174/156802611795371332. 21050177 10.2174/156802611795371332 PMC3725992

[21] Ma Y, Wink M. Lobeline, a piperidine alkaloid from Lobelia can reverse P-gp dependent multidrug resistance in tumor cells. Phytomedicine. 2008;15:754–8. 10.1016/j.phymed.2007.11.028. 18222670 10.1016/j.phymed.2007.11.028

[22] Zhao M, Zhou L, Zhang Q, Wang M, Dong Y, Wang Y, et al. Targeting MAPK14 by lobeline upregulates slurp1‐mediated inhibition of alternative activation of TAM and retards colorectal cancer growth. Adv Sci. 2025;12:2407900. 10.1002/advs.202407900. 10.1002/advs.202407900 PMC11904982 39840525

[23] Chang WH, Cerione RA, Antonyak MA. Extracellular vesicles and their roles in cancer progression. Methods Mol Biol. 2021;2174:143–70. 10.1007/978-1-0716-0759-6_10. 32813249 10.1007/978-1-0716-0759-6_10 PMC8008708

[24] Kalluri R, McAndrews KM. The role of extracellular vesicles in cancer. Cell. 2023;186:1610–26. 10.1016/j.cell.2023.03.010. 37059067 10.1016/j.cell.2023.03.010 PMC10484374

[25] King HW, Michael MZ, Gleadle JM. Hypoxic enhancement of exosome release by breast cancer cells. BMC Cancer. 2012;12:421. 10.1186/1471-2407-12-421. 22998595 10.1186/1471-2407-12-421 PMC3488584

[26] Zhang W, Zhou X, Yao Q, Liu Y, Zhang H, Dong Z. HIF-1-mediated production of exosomes during hypoxia is protective in renal tubular cells. Am J Physiol Ren Physiol. 2017;313:906. 10.1152/ajprenal.00178.2017. 10.1152/ajprenal.00178.2017 PMC5668579 28679592

[27] Patton MC, Zubair H, Khan MA, Singh S, Singh AP. Hypoxia alters the release and size distribution of extracellular vesicles in pancreatic cancer cells to support their adaptive survival. J Cell Biochem. 2020;121:828–39. 10.1002/jcb.29328. 31407387 10.1002/jcb.29328 PMC6878126

[28] Wang X, Wu R, Zhai P, Liu Z, Xia R, Zhang Z, et al. Hypoxia promotes EV secretion by impairing lysosomal homeostasis in HNSCC through negative regulation of ATP6V1A by HIF-1α. J Extracell Vesicles. 2023;12:12310. 10.1002/jev2.12310. 10.1002/jev2.12310 PMC9903130 36748335

[29] Li Y, Tan J, Miao Y, Zhang Q. MicroRNA in extracellular vesicles regulates inflammation through macrophages under hypoxia. Cell Death Discov. 2021;7:285. 10.1038/s41420-021-00670-2. 34635652 10.1038/s41420-021-00670-2 PMC8505641

[30] Kothandan VK, Kothandan S, Kim DH, Byun Y, Lee YK, Park IK, et al. Crosstalk between stress granules, exosomes, tumour antigens, and immune cells: significance for cancer immunity. Vaccines. 2020;8. 10.3390/vaccines8020172. 10.3390/vaccines8020172 PMC7349635 32276342

[31] Somasekharan SP, El-Naggar A, Leprivier G, Cheng H, Hajee S, Grunewald TG, et al. YB-1 regulates stress granule formation and tumor progression by translationally activating G3BP1. J Cell Biol. 2015;208:913–29. 10.1083/jcb.201411047. 25800057 10.1083/jcb.201411047 PMC4384734

[32] Lyons SM, Achorn C, Kedersha NL, Anderson PJ, Ivanov P. YB-1 regulates tiRNA-induced stress granule formation but not translational repression. Nucleic Acids Res. 2016;44:6949–60. 10.1093/nar/gkw418. 27174937 10.1093/nar/gkw418 PMC5001593

[33] Suresh PS, Tsutsumi R, Venkatesh T. YBX1 at the crossroads of non-coding transcriptome, exosomal, and cytoplasmic granular signaling. Eur J Cell Biol. 2018;97:163–7. 10.1016/j.ejcb.2018.02.003. 29478751 10.1016/j.ejcb.2018.02.003

[34] Sfakianos AP, Mellor LE, Pang YF, Kritsiligkou P, Needs H, Abou-Hamdan H, et al. The mTOR-S6 kinase pathway promotes stress granule assembly. Cell Death Differ. 2018;25:1766–80. 10.1038/s41418-018-0076-9. 29523872 10.1038/s41418-018-0076-9 PMC6004310

[35] Seguin SJ, Morelli FF, Vinet J, Amore D, De Biasi S, Poletti A, et al. Inhibition of autophagy, lysosome and VCP function impairs stress granule assembly. Cell Death Differ. 2014;21:1838–51. 10.1038/cdd.2014.103. 25034784 10.1038/cdd.2014.103 PMC4227144

[36] Buchan JR, Kolaitis RM, Taylor JP, Parker RX. Eukaryotic stress granules are cleared by autophagy and Cdc48/VCP function. Cell. 2013;153:1461–74. 10.1016/j.cell.2013.05.037. 23791177 10.1016/j.cell.2013.05.037 PMC3760148

[37] Teng LH, Crooks PA, Dwoskin LP. Lobeline displaces [3H] dihydrotetrabenazine binding and releases [3H]dopamine from rat striatal synaptic vesicles: comparison with d- amphetamine. J Neurochem. 1998;71:258–65. 10.1046/j.1471-4159.1998.71010258.x. 9648873 10.1046/j.1471-4159.1998.71010258.x

[38] Teng L, Crooks PA, Sonsalla PK, Dwoskin LP. Lobeline and nicotine evoke [3H]overflow from rat striatal slices preloaded with [3H]dopamine: differential inhibition of synaptosomal and vesicular [3H]dopamine uptake. J Pharmacol Exp Ther. 1997;280:1432–44. 9067333

[39] Dwoskin LP, Crooks PA. A novel mechanism of action and potential use for lobeline as a treatment for psychostimulant abuse. Biochem Pharm. 2002;63:89–98. 10.1016/S0006-2952(01)00899-1. 11841781 10.1016/s0006-2952(01)00899-1

[40] Shurtleff MJ, Yao J, Qin Y, Nottingham RM, Temoche-Diaz MM, Schekman R, et al. Broad role for YBX1 in defining the small noncoding RNA composition of exosomes. Proc Natl Acad Sci USA. 2017;114:8987. 10.1073/pnas.1712108114. 10.1073/pnas.1712108114 PMC5663387 29073095

[41] Chiba M, Miyata K, Okawa H, Tanaka Y, Ueda K, Seimiya H, et al. YBX1 regulates satellite II RNA loading into small extracellular vesicles and promotes the senescent phenotype. Int J Mol Sci. 2023;24. 10.3390/ijms242216399. 10.3390/ijms242216399 PMC10671301 38003589

[42] Li T, Zeng Z, Fan C, Xiong W. Role of stress granules in tumorigenesis and cancer therapy. Biochim Biophys Acta Rev Cancer. 2023;1878:189006. 10.1016/j.bbcan.2023.189006. 37913942 10.1016/j.bbcan.2023.189006

[43] Ogunrinu TA, Sontheimer H. Hypoxia increases the dependence of glioma cells on glutathione. J Biol Chem. 2010;285:37716–24. 10.1074/jbc.M110.161190. 20858898 10.1074/jbc.M110.161190 PMC2988376

[44] Liu-Yesucevitz L, Bilgutay A, Zhang YJ, Vanderweyde T, Citro A, Mehta T, et al. Tar DNA binding protein-43 (TDP-43) associates with stress granules: analysis of cultured cells and pathological brain tissue. PLoS ONE. 2010;5:13250. 10.1371/journal.pone.0013250. 10.1371/journal.pone.0013250 PMC2952586 20948999

[45] Bosco DA, Lemay N, Ko HK, Zhou H, Burke C, Kwiatkowski TJ Jr, et al. Mutant FUS proteins that cause amyotrophic lateral sclerosis incorporate into stress granules. Hum Mol Genet. 2010;19:4160–75. 10.1093/hmg/ddq335. 20699327 10.1093/hmg/ddq335 PMC2981014

[46] Wolozin B. Regulated protein aggregation: stress granules and neurodegeneration. Mol Neurodegener. 2012;7:56. 10.1186/1750-1326-7-56. 23164372 10.1186/1750-1326-7-56 PMC3519755

[47] Arimoto K, Fukuda H, Imajoh-Ohmi S, Saito H, Takekawa M. Formation of stress granules inhibits apoptosis by suppressing stress-responsive MAPK pathways. Nat Cell Biol. 2008;10:1324–32. 10.1038/ncb1791. 18836437 10.1038/ncb1791

[48] Fujikawa D, Nakamura T, Yoshioka D, Li Z, Moriizumi H, Taguchi M, et al. Stress granule formation inhibits stress-induced apoptosis by selectively sequestering executioner caspases. Curr Biol. 2023;33:1967–81. 10.1016/j.cub.2023.04.012. 37119817 10.1016/j.cub.2023.04.012

[49] Glauninger H, Wong Hickernell CJ, Bard JAM, Drummond DA. Stressful steps: progress and challenges in understanding stress-induced mRNA condensation and accumulation in stress granules. Mol Cell. 2022;82:2544–56. 10.1016/j.molcel.2022.05.014. 35662398 10.1016/j.molcel.2022.05.014 PMC9308734

[50] Marmor-Kollet H, Siany A, Kedersha N, Knafo N, Rivkin N, Danino YM, et al. Spatiotemporal proteomic analysis of stress granule disassembly using APEX reveals regulation by SUMOylation and links to ALS pathogenesis. Mol Cell. 2020;80:876–91. 10.1016/j.molcel.2020.10.032. 33217318 10.1016/j.molcel.2020.10.032 PMC7816607

[51] Keiten-Schmitz J, Wagner K, Piller T, Kaulich M, Alberti S, Müller S. The nuclear SUMO-targeted ubiquitin quality control network regulates the dynamics of cytoplasmic stress granules. Mol Cell. 2020;79:54–67. 10.1016/j.molcel.2020.05.017. 32521226 10.1016/j.molcel.2020.05.017

[52] Eldh M, Ekström K, Valadi H, Sjöstrand M, Olsson B, Jernås M, et al. Exosomes communicate protective messages during oxidative stress; possible role of exosomal shuttle RNA. PLoS ONE. 2010;5:15353. 10.1371/journal.pone.0015353. 10.1371/journal.pone.0015353 PMC3003701 21179422

[53] Maacha S, Bhat AA, Jimenez L, Raza A, Haris M, Uddin S, et al. Extracellular vesicles-mediated intercellular communication: roles in the tumor microenvironment and anti-cancer drug resistance. Mol Cancer. 2019;18:55. 10.1186/s12943-019-0965-7. 30925923 10.1186/s12943-019-0965-7 PMC6441157

[54] Eitan E, Suire C, Zhang S, Mattson MP. Impact of lysosome status on extracellular vesicle content and release. Ageing Res Rev. 2016;32:65–74. 10.1016/j.arr.2016.05.001. 27238186 10.1016/j.arr.2016.05.001 PMC5154730

[55] Xie Y, Bergström T, Jiang Y, Johansson P, Marinescu VD, Lindberg N, et al. The human glioblastoma cell culture resource: validated cell models representing all molecular subtypes. EBioMedicine. 2015;2:1351–63. 10.1016/j.ebiom.2015.08.026. 26629530 10.1016/j.ebiom.2015.08.026 PMC4634360

