# Supplementary figures and images for: Modulation of stress granules by lobeline increases cell death in hypoxia and impacts the ability of glioblastoma cells to secrete extracellular vesicles
Source: Cell Death Discov. 2025 Oct 6;11:432. doi: 10.1038/s41420-025-02692-6 (PMC12501001; doi:10.1038/s41420-025-02692-6)

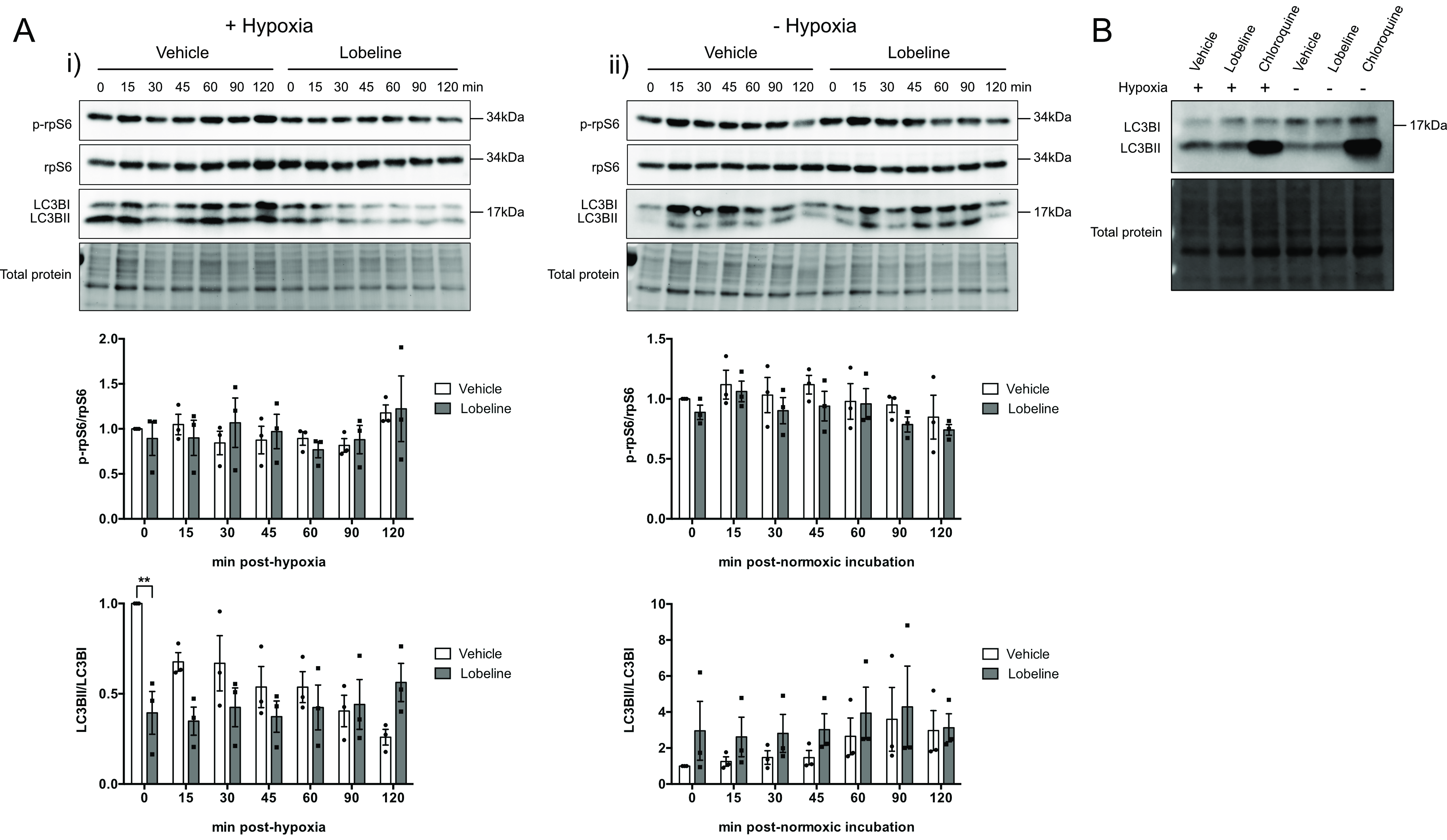

Supplement: Supplementary file 1 — Supplementary Figure 1 [file 41420_2025_2692_MOESM1_ESM.tif]

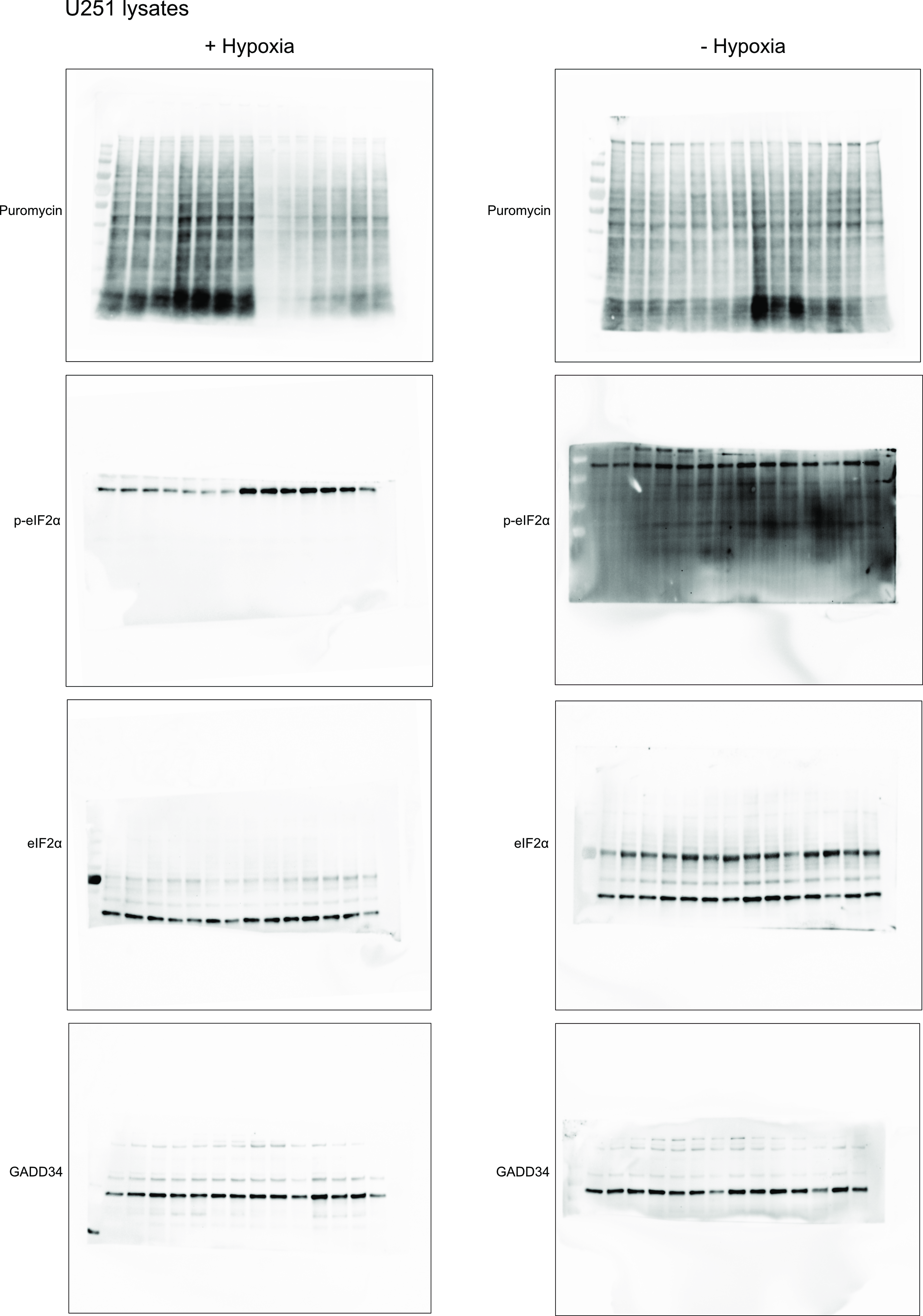

Supplement: Supplementary file 4 — Uncropped Westerns [file 41420_2025_2692_MOESM4_ESM.tif]

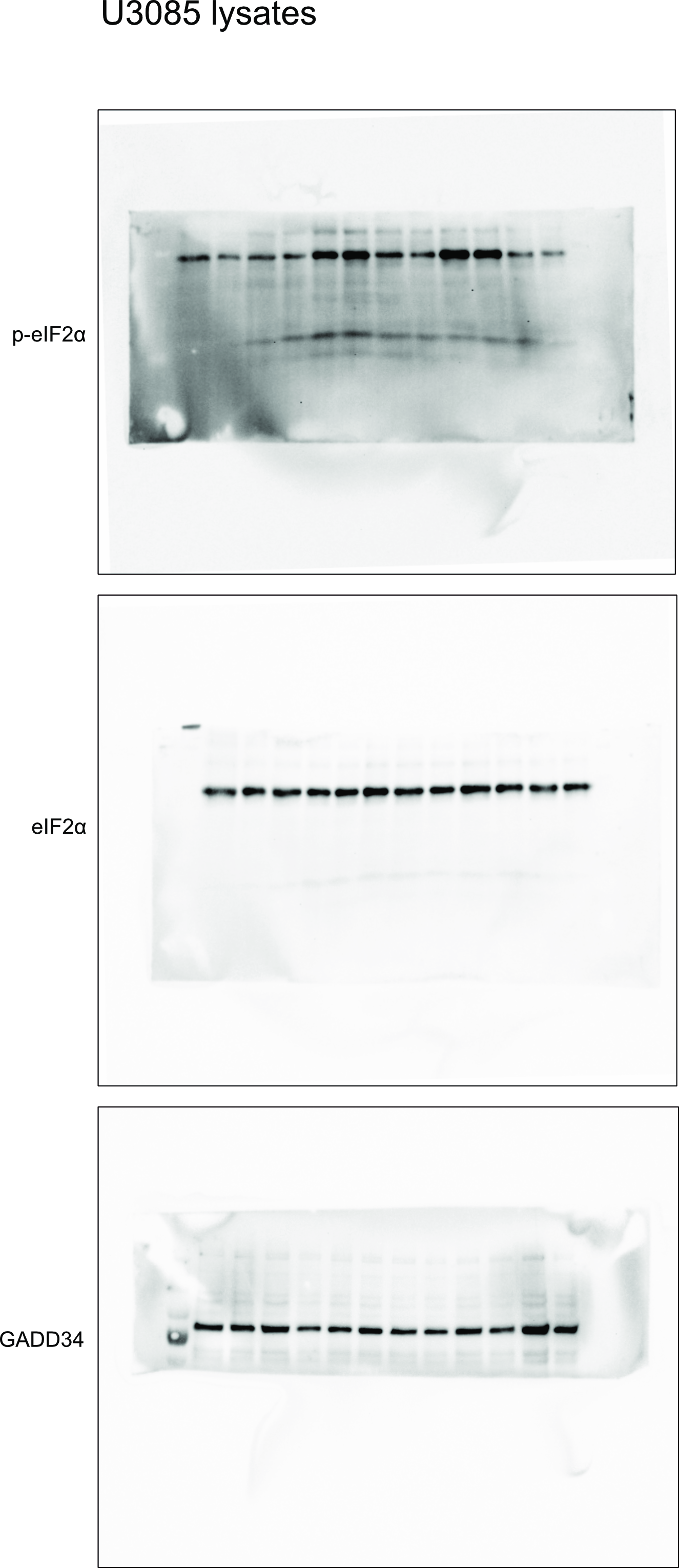

Supplement: Supplementary file 5 — Uncropped Westerns [file 41420_2025_2692_MOESM5_ESM.tif]

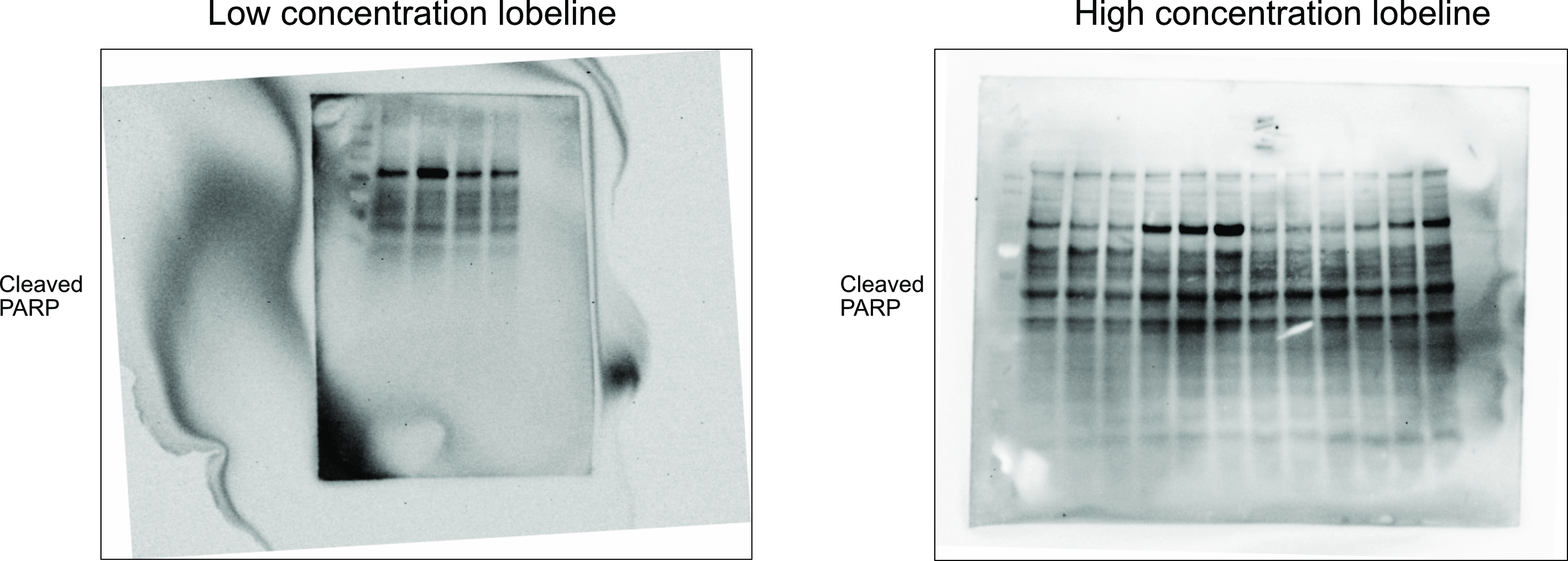

Supplement: Supplementary file 6 — Uncropped Westerns [file 41420_2025_2692_MOESM6_ESM.tif]

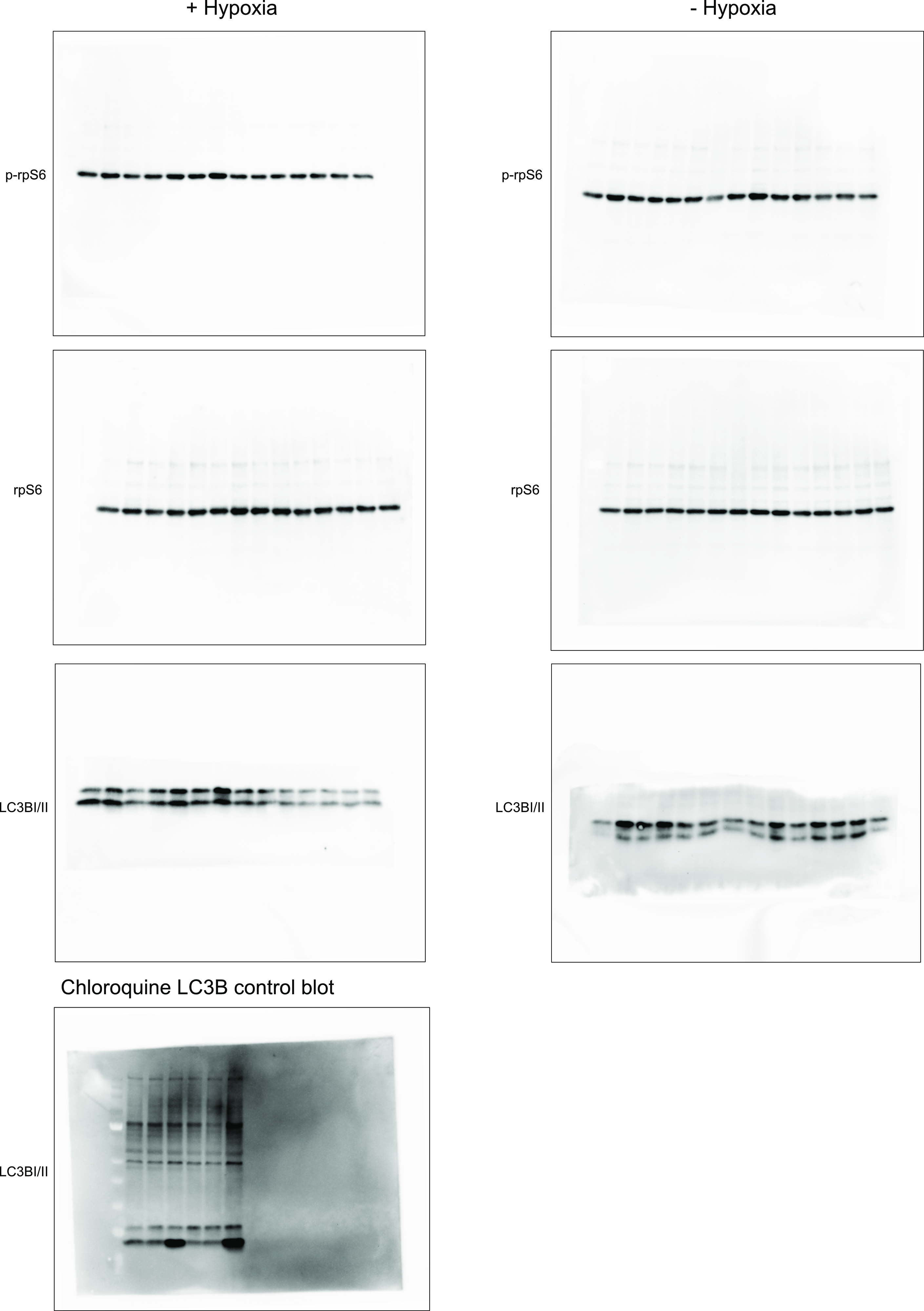

Supplement: Supplementary file 7 — Uncropped Westerns [file 41420_2025_2692_MOESM7_ESM.tif]
