# Supplementary material for: Modulation of stress granules by lobeline increases cell death in hypoxia and impacts the ability of glioblastoma cells to secrete extracellular vesicles
Source: Cell Death Discov. 2025 Oct 6;11:432. doi: 10.1038/s41420-025-02692-6 (PMC12501001; doi:10.1038/s41420-025-02692-6)
Supplement: Supplementary file 2 — Supplementary Figure 1 Legend [file 41420_2025_2692_MOESM2_ESM.docx]

**Supplemental Figure 1. Lobeline treatment does not lead to changes in mTOR signaling or autophagy.** (**A**) U251 cells were treated with 50 μM lobeline or vehicle control for 1 h prior to a 2 h incubation ± hypoxia (<1% O_2_). At various times post-hypoxic (i) or corresponding normoxic (ii) incubation (0-120 min) total cell lysates were harvested and probed for total rpS6, phospho-rpS6 and LC3B. All blots were normalized to their respective total lane protein and are represented as ratios with vehicle control T = 0 min normalized to 1. The level of rpS6 phosphorylation is presented as the ratio of phospho-rpS6 to total rpS6. LC3B is presented as a ratio of LC3BII (bottom band) to LC3BI (top band). Data is presented as the mean of biological replicates (N=3) ± SEM, two-way ANOVA, Sidak’s multiple comparisons test **p<0.01 (**B**) U251 cells were treated with 50 μM lobeline, 40 μM chloroquine or vehicle control for 1 h prior to a 2 h incubation ± hypoxia (<1% O_2_). Total cell lysates were harvested 60 min post-hypoxic or corresponding normoxic incubation and probed for LC3B.
